# Intelligent Hydrocolloid-Based Delivery Systems: Innovations in Pharmacy and Cosmetics

**DOI:** 10.3390/molecules31142468

**Published:** 2026-07-15

**Authors:** Karen Khachatryan, Oskar Michalski, Klaudia Michalska

**Affiliations:** 1 Laboratory of Nanotechnology and Nanomaterials, Faculty of Food Technology, University of Agriculture in Krakow, Al. Mickiewicza 21, 31-120 Krakow, Poland; 2 Department of Chemistry, Faculty of Food Technology, University of Agriculture in Krakow, Al. Mickiewicza 21, 31-120 Krakow, Poland; oskar.michalski@urk.edu.pl; 3 Department of Phytochemistry, Maj Institute of Pharmacology, Polish Academy of Sciences, Smętna Street 12, 31-343 Krakow, Poland; klaudia.michalska@if-pan.edu.pl

**Keywords:** hydrocolloid-based delivery systems, natural polysaccharides, hybrid hydrogels, stimuli-responsive release, oral delivery systems, transdermal patches, injectable depots, wound dressings, cosmeceuticals, 3D bioprinting, theranostic smart patches

## Abstract

Hydrocolloid-based and hydrocolloid-dominant hybrid matrices have developed from conventional rheology modifiers into functional platforms for pharmaceutical and cosmetic delivery. This review focuses on systems in which hydrocolloid chemistry, hydration, ionisation, bioadhesion, and network architecture determine swelling, mechanical behaviour, biocompatibility, and controlled release. The discussion covers alginate, chitosan, hyaluronic acid, pectin, carrageenan, dextran, gellan gum, collagen, cellulose derivatives, and selected hybrid architectures in which synthetic or semi-synthetic components provide a defined responsive function. Rather than treating all smart polymers as a single class, the review compares how pH, enzymatic, redox/ROS, thermo-responsive, magnetic, optical, ultrasound-mediated, and multi-trigger mechanisms operate within hydrocolloid-rich matrices. Pharmaceutical examples are considered across oral, transdermal, injectable depot, wound-healing, and regenerative applications, while cosmetic and cosmeceutical systems are discussed in relation to active stabilisation, dermal residence, barrier support, and personalised skincare. By linking material class, trigger mechanism, route of administration, and translational constraints, the review identifies the main advantages of hydrocolloids as delivery matrices as well as their current limitations, including burst release, modest mechanical strength, hydrophobic-drug loading challenges, sterilisation sensitivity, source variability, and regulatory complexity.

## 1. Introduction

Hydrocolloids and the hydrogels prepared from them form a versatile class of hydrated, semi-solid polymer networks with a high water content, forming three-dimensional (3D) networks of hydrophilic chains [1,2]. In terms of physical, rheological, and mechanical properties, they can reproduce selected extracellular-matrix-like features of living tissues, particularly hydration, softness, and diffusive transport [3]. The broader hydrogel field entered modern biomedical applications in 1960, when O. Wichterle and D. Lim described a synthetic hydrogel based on poly(2-hydroxyethyl methacrylate) (pHEMA), initially intended for the production of soft contact lenses [4]. Since then, hydrogels and hydrocolloid-derived matrices have become important platforms for therapeutic innovation [5]. Their ability to retain large amounts of physiological fluids without dissolving, resulting from the presence of numerous hydrophilic groups, has made them an important component of modern tissue engineering and delivery systems [6].

Beyond their pharmaceutical role, hydrocolloids are also central to modern cosmetology and cosmeceutical design. Traditionally used as thickeners, stabilisers, and rheology modifiers, they now increasingly serve as functional matrices for the stabilisation of labile actives, prolonged skin residence, controlled dermal delivery, and barrier-support formulations [7]. This transition is particularly evident for polysaccharides such as xanthan, carrageenan, alginate, cellulose derivatives, chitosan, and hyaluronic acid, which combine favourable formulation properties with growing relevance in advanced skincare and personalised cosmetic systems [8,9,10,11]. In this review, semi-synthetic and synthetic components are considered only when they form hydrocolloid-dominant hybrid architectures or provide a necessary comparison for hydrocolloid-based delivery. For decades, macromolecular biopolymers—such as gelatine, carrageenan, xanthan gum, or cellulose derivatives—served purely passive and structural functions in pharmacy and cosmetology [12]. They were primarily used as inexpensive thickeners, viscosity modifiers, or stabilisers preventing the syneresis of O/W and W/O emulsions [8]. However, with the rapid development of materials engineering, polymer chemistry, nanostructured carriers, and advanced cross-linking strategies, many hydrocolloid-based systems have evolved from passive excipients into functional and, in some cases, stimuli-responsive delivery platforms [9,13,14].

**Figure 1:**
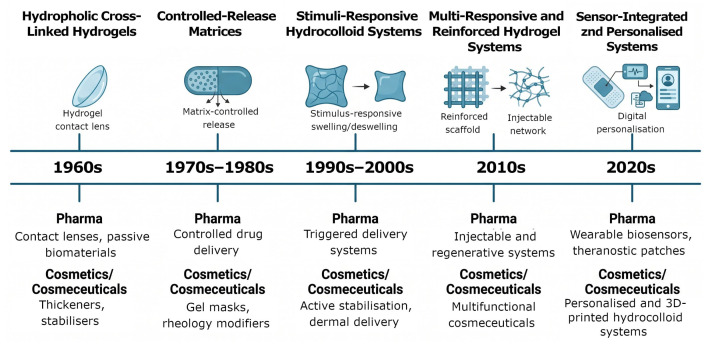
Timeline of the evolution of hydrocolloid-based systems in pharmaceutical and cosmetic applications, illustrating the progression from early hydrophilic cross-linked hydrogels and controlled-release matrices to stimuli-responsive, reinforced, personalised and sensor-integrated systems. Representative pharmaceutical and cosmetic/cosmeceutical applications associated with each developmental stage are indicated schematically.

A schematic timeline (Figure 1) illustrates the historical progression of hydrocolloid-based systems from early hydrophilic cross-linked hydrogels and controlled-release matrices to stimuli-responsive, reinforced, and sensor-integrated personalised platforms. In the context of the present review, this progression should be understood not only as a general increase in material complexity but also as an expansion of hydrocolloid functionality across both pharmaceutical and cosmetic applications, including active stabilisation, controlled dermal delivery, barrier-support systems, and personalised skin-contact formats [10,11].

This review therefore addresses hydrocolloid-based and hydrocolloid-dominant hybrid delivery systems, with emphasis on how material chemistry and network design translate into delivery performance. Section 2 summarises the principal hydrocolloid classes and the material properties that govern their performance. Section 3 links these properties to defined trigger mechanisms and release principles. Section 4 maps specific hydrocolloid classes onto major pharmaceutical applications, including oral, transdermal, injectable, wound-healing, and regenerative systems. Section 5 extends the discussion to cosmetic and cosmeceutical formulations, including the stabilisation of labile actives, controlled dermal delivery, barrier-support skincare, and personalised formats such as 3D-printed masks. The Section 6 critically discusses translational barriers, regulatory constraints, and the convergence of pharmaceutical and cosmetic technologies [15,16,17,18,19].

## 2. Hydrocolloid Classes and Design-Relevant Functional Properties

Designing effective delivery systems for active pharmaceutical ingredients (APIs) requires an understanding of polymer chemistry, network topology and their relationship to functional performance [20]. Hydrocolloids can be classified by origin, chemical composition, ionic character, gelling behaviour and application profile, but none of these descriptors alone predicts delivery performance [8,21,22].

### 2.1. Classification by Origin

#### 2.1.1. Algal and Marine Origin

Representative polymers in this group include alginic acid and its salts isolated from the cell walls of brown algae [23,24]. Alginates, rich in blocks of guluronic and mannuronic acid, exhibit outstanding gelling properties in the presence of divalent cations and are widely used in microencapsulation, oral protection and hydrogel-based delivery systems [23,24,25,26,27]. Fucoidan, a highly sulphated marine polysaccharide, has attracted increasing interest because of its native antioxidant, anti-tumour and antithrombotic properties, as well as its potential role in localised hydrogel-based therapy [9,28,29,30]. Microalgae extracts are also an excellent source of mycosporine-like amino acids (MAAs), which form the basis for modern UV-protective cosmetic strategies [28,29].

#### 2.1.2. Microbial, Animal, and Plant Origin

Bacterial exopolysaccharides (EPS), such as xanthan gum, gellan gum, and dextran, are particularly valuable because their biosynthetic origin enables reproducible chemistry, high hydration capacity, and pronounced rheological functionality [31,32,33,34,35]. Xanthan gum provides pseudoplastic flow and formulation stability, gellan gum forms ion-sensitive gels with ophthalmic and injectable potential, and dextran is readily modified into degradable conjugates and dynamic networks for injectable or anti-tumour systems [31,32,33,34,35,36,37,38,39].

Structurally characterised fungal polysaccharides can also serve as useful comparison points for molecular-design discussion. For example, a recent Grifola frondosa polysaccharide study links molecular-weight distribution, beta-glucan branching and glycosidic linkage pattern with biological activity; however, such examples should be distinguished from formulated hydrocolloid delivery matrices unless they are used directly as carrier materials [40].

Animal-derived hydrocolloids complement these materials by contributing native bioactivity. Chitosan, obtained by deacetylation of chitin, is the best-known cationic polysaccharide, with intrinsic mucoadhesive, haemostatic, and antimicrobial properties that are especially useful in wound care, mucosal delivery, and injectable matrices [36,41,42,43,44,45,46]. Collagen and hyaluronic acid (HA), in turn, more closely resemble the extracellular matrix of skin and connective tissues, which explains their importance in regenerative medicine, dermal repair, and soft biomaterials for cell-supportive delivery [37,47,48,49,50,51,52].

Plant-derived hydrocolloids extend this functional spectrum by offering a wide range of physicochemical properties linked to their structural diversity. Modified cellulose derivatives (e.g., CMC, HPMC, and CNC-containing systems) are widely used as matrix-forming excipients and controlled-release scaffolds because they combine swelling capacity with robust processing properties [53,54,55,56,57,58]. Pectin, rich in galacturonic acid units, forms pH-sensitive gels via ionic cross-linking, while locust bean gum (LBG), a galactomannan, exhibits resistance to enzymatic degradation in the upper gastrointestinal tract and undergoes selective fermentation in the colon [13,59,60,61]. This makes plant polysaccharides particularly attractive for site-specific gastrointestinal delivery and colon-targeted systems [13,59,60,61,62,63].

Together, microbial, animal, and plant hydrocolloids provide complementary functionalities spanning rheology control, bioadhesion, ionotropic gelation, enzymatic degradability, and site-specific delivery, while also underpinning newer injectable and targeted-delivery strategies based on polysaccharide-rich hydrogel matrices [14,22,64]. Although the present review is focused primarily on natural hydrocolloids, selected synthetic or semi-synthetic polymers are referred to later only where they provide comparative context or form part of hybrid architectures directly relevant to hydrocolloid-based delivery systems.

The classification and key functional properties of these hydrocolloids are summarised in Table 1.

For the hydrocolloids listed in Table 1, gelation, swelling and network mechanics are best treated as formulation-dependent descriptors rather than fixed material constants. Alginate, pectin, carrageenan and gellan gum provide typical examples of ionotropic or ion-strengthened networks; in these systems, the cation species, cation concentration and junction density determine swelling pressure and modulus. Chitosan and hyaluronic acid are more strongly affected by pH-dependent ionisation and cross-linking density, which influence mucoadhesion, water uptake, viscoelasticity, injectability and residence time. Dextran is often modified into dynamic imine, hydrazone, boronate or redox-cleavable networks, so degradation-controlled softening becomes part of the release mechanism. Cellulose derivatives and LBG usually act through hydration, chain entanglement and gel-layer formation, whereas collagen forms ECM-like fibrillar networks whose mechanics depend on concentration, fibrillogenesis and cross-linking [23,24,25,26,27,32,33,34,38,39,50,54,55,56,60,63,66,67,82,83,86,87,88,89,90,91].

### 2.2. Critical Properties: Thermodynamics of Gelation, Swelling, and Network Mechanics

Cross-linking reactions define not only the chemical structure of hydrocolloid networks but also their thermodynamic stability and mechanical performance, which directly govern drug release behaviour [22,39,64,86]. Physical cross-linking is based on reversible non-covalent interactions (e.g., hydrogen bonding, ionic interactions, hydrophobic associations), whereas chemical cross-linking introduces permanent covalent junctions that increase network integrity and mechanical strength. From a thermodynamic perspective, gelation reflects the formation of a three-dimensional network that minimises the free energy of the system through a balance of polymer–polymer and polymer–solvent interactions.

Swelling is governed by the equilibrium between the osmotic pressure driving solvent uptake and the elastic retractive forces of the polymer network, as described in hydrogel swelling and transport models [63,88,89]. The degree of swelling therefore depends on parameters such as cross-linking density, polymer–solvent affinity, charge density and the presence of ionisable groups. Recent studies on hydrogel-based delivery systems further confirm that cross-linking parameters and matrix composition strongly affect rheological behaviour, release kinetics and biological performance in topical and injectable applications [50,87]. Polyelectrolyte hydrocolloid networks, containing a high density of charged moieties, may exhibit enhanced swelling due to electrostatic repulsion and Donnan osmotic effects, which in turn can accelerate diffusion of encapsulated hydrophilic molecules [63,88,89].

**Figure 2:**
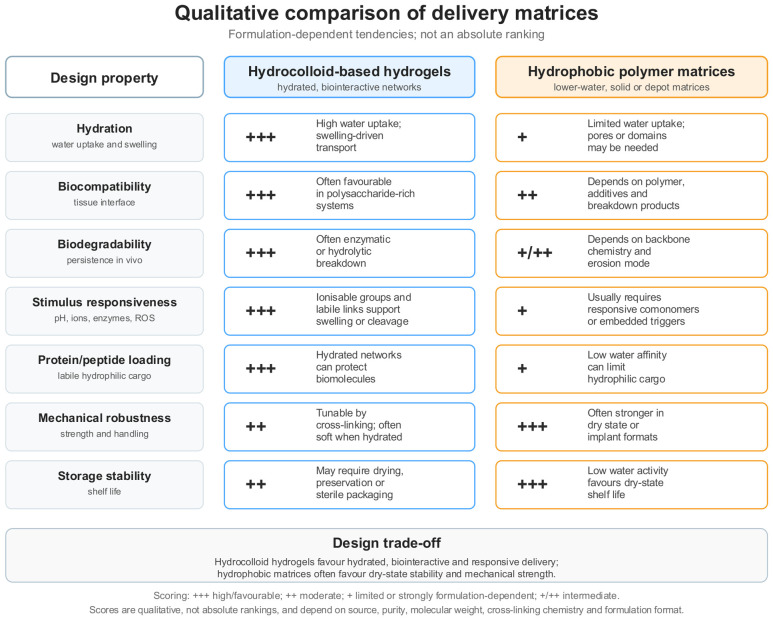
Functional comparison of hydrocolloid-based hydrogels and hydrophobic polymer matrices relevant to delivery-system design. The colour coding separates the two matrix classes and is used only as a visual guide; the qualitative assessment is expressed by the number of plus signs. The comparison is intended as a design-oriented summary rather than an absolute ranking, because each property depends on polymer source, purity, molecular weight, cross-linking chemistry, additives, processing, and formulation format.

From a mechanical standpoint, the elastic modulus of a hydrogel is directly related to the density and functionality of network junctions. This relationship is particularly relevant in HA-based and hybrid hydrogel systems, where tuning cross-linker architecture may simultaneously influence stiffness, swelling, injectability, and in-use stability [50,87]. Increasing cross-linking density generally enhances stiffness and reduces swelling, while loosely cross-linked networks exhibit higher deformability and permeability. Recent developments, including “click” chemistry (e.g., Diels–Alder reactions) and dynamic covalent bonds such as Schiff bases, enable the design of adaptive and self-healing hydrogels, in which reversible network rearrangements allow recovery after mechanical damage while maintaining structural integrity [92]. A schematic comparison of selected functional properties of hydrocolloid-based hydrogels and hydrophobic polymer matrices relevant to delivery-system design is presented in Figure 2. These material differences are not merely descriptive. They decide whether a hydrocolloid is better suited to a protective oral matrix, a mucoadhesive carrier, an injectable depot, a wound dressing or a dermal/cosmetic hydrogel. In most formulations, higher ionic or covalent cross-link density strengthens the network and slows release, whereas higher charge density, lower junction density or trigger-induced bond cleavage increase swelling, mesh size and permeability [50,63,86,87,88,89,90,91].

Hydrocolloid-based hydrogels score highly for hydration, biocompatibility, biodegradability, responsiveness and protein/peptide loading mainly because their backbones are hydrophilic and often ionisable. Carboxyl, hydroxyl, amino and sulphate groups support water uptake, pH- or ion-dependent swelling and a hydrated microenvironment suitable for labile biomolecules. Hydrophobic polymeric matrices show a different profile. Their lower water content can improve storage stability and mechanical robustness by limiting hydrolysis, microbial risk and premature diffusion, but it may also make hydrophilic drugs, proteins or peptides more difficult to load without additional formulation strategies. Figure 2 therefore compares two useful but different design spaces rather than ranking one material family as universally superior.

### 2.3. Characterisation Toolbox for Hydrocolloid Delivery Systems

Because hydrocolloid performance is strongly formulation-dependent, comparative assessment should combine structural, physicochemical, mechanical, release and biological tests. Rheology is needed to define sol–gel transitions, injectability, thixotropy, and storage/loss moduli; swelling and degradation assays quantify water uptake, erosion, and matrix stability under simulated gastric, intestinal, wound or skin conditions; SEM or cryo-SEM can visualise pore structure after careful sample preparation; FTIR, NMR or re-lated spectroscopic methods can verify functional groups, cross-linking and drug-polymer interactions; tensile, compression, puncture or adhesion tests define mechanical suitability for patches, depots and wound dressings; in vitro release models should be fitted to diffu-sion-, relaxation- or erosion-controlled kinetics; and cytocompatibility, irritation, haemo-compatibility and microbiological tests should be selected according to the intended route of administration. This characterisation toolbox also helps distinguish genuine stimulus-responsiveness from simple hydration-driven release.

### 2.4. Biocompatibility and “Green” Engineering

Natural biopolymers often offer important advantages in terms of biocompatibility and sustainability, although these properties depend on source, purity, chemical modification, and the intended route of administration [93]. Many hydrocolloids used in pharmaceutical and cosmetic formulations have a favourable safety profile and, in selected cases, Generally Recognised As Safe (GRAS) status or comparable regulatory familiarity [94]. Their susceptibility to enzymatic or hydrolytic degradation may reduce long-term persistence in vivo, but degradation behaviour must be assessed individually for each material and formulation [95,96]. From a sustainability perspective, current hydrogel design increasingly considers not only source renewability but also processing requirements, degradation behaviour, and the overall formulation burden associated with translational use [97].

## 3. Stimuli-Responsive Mechanisms in Hydrocolloid-Dominant Systems

In hydrocolloid-based delivery systems, stimulus responsiveness is useful only when the trigger produces a predictable change in matrix hydration, charge state, bond stability, permeability, or erosion while the hydrocolloid component remains central to the formulation [12,15,22,98,99,100]. Unlike passive carriers, these systems enable controlled, spatially and temporally modulated release—through changes in ionisation, bond stability, permeability, swelling, or matrix integrity, thereby supporting more selective and application-specific delivery.

Release mechanisms vary with material architecture and conditions [88,101]. In simple systems, drug diffuses from a polymer matrix or degrades with the carrier. Advanced systems respond to stimuli—pH, temperature, enzymes—triggering structural reorganisation and selective release [102]. In situ forming hydrogels are administered as low-viscosity solutions and gel at the application site, creating a long-acting depot with near-zero-order kinetics [17,103,104]. The choice of hydrocolloid and responsiveness mechanism depends on the therapeutic goal, such as tumour-targeted release or chronic wound management.

**Figure 3:**
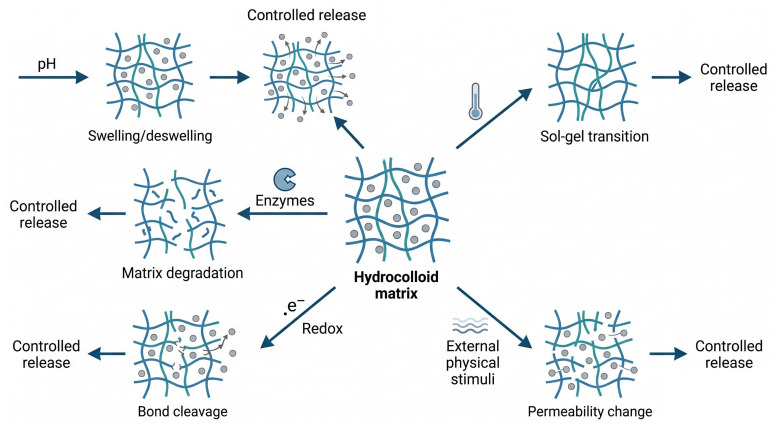
Schematic representation of the principal stimulus-responsive pathways relevant to hydrocolloid-based delivery systems. Depending on trigger type, the hydrocolloid matrix undergoes swelling/deswelling, matrix degradation, bond cleavage, sol–gel transition, or permeability change, which ultimately modulates controlled release.

Hydrocolloids respond to chemical, biological and physical stimuli. Effective design requires balancing material chemistry, network architecture, mass transport, and the trade-off between stability and sensitivity [105]. As summarised in Figure 3, these trigger classes do not act at the same structural level: some primarily alter charge density and swelling, others induce bond cleavage or matrix degradation, while externally applied stimuli more often change permeability, local temperature, or network relaxation. In each case, the final pharmaceutical consequence is a change in mesh size, diffusional resistance, or matrix erosion, which then determines cargo release.

The principal stimulus-responsive mechanisms relevant to hydrocolloid-based delivery systems are summarised in Figure 3.

### 3.1. Trigger Mechanisms in Stimuli-Responsive Hydrocolloids

Trigger mechanisms are classified by signal nature: endogenous biochemical cues, externally applied physical stimuli, and multi-responsive platforms.

The practical value of each trigger type differs substantially. pH-responsive systems are simple, formulation-friendly and useful for gastrointestinal or wound gradients, but pH alone may lack disease specificity. Enzyme-responsive matrices can be more selective, although release depends on enzyme expression, local diffusion and patient-to-patient variability. ROS/redox systems match inflammatory or tumour-associated chemistry but require careful control of premature oxidation or reduction. Thermo-responsive systems are attractive for injectable depots and local retention, yet they often rely on synthetic or semi-synthetic components and must be checked for gelation temperature, reversibility and dilution effects. Magnetic, light- and ultrasound-triggered systems offer spatial control, but they add hardware, penetration depth, heating and safety constraints. Multi-responsive systems can improve selectivity, but they also increase manufacturing and regulatory complexity.

#### 3.1.1. pH-Responsive Systems

pH-responsive systems are well-established internally activated carriers [20,90,106,107,108,109,110]. Physiological and pathological environments exhibit predictable pH differences, including the gastrointestinal gradient, tumour acidity and inflamed or infected wound microenvironments. In hydrocolloid-based systems, the mechanism relies mainly on ionisable groups, such as carboxyl and amino functionalities, present in alginate, pectin, chitosan, hyaluronic acid derivatives, or modified cellulose-based matrices. Changes in pH cause protonation or deprotonation, altering fixed charge density, osmotic pressure, and polymer–polymer interactions, which in turn affect swelling, permeability or network destabilisation [20,90,107,108,109,110].

Acidic polymers swell in alkali; basic polymers swell in acid. Amphoteric systems swell at both extremes but collapse near the isoelectric point. pH-responsiveness can be implemented in cross-linked hydrogels (reversible swelling modulating diffusion) or self-assembling systems (disintegration and rapid release). Alternatively, dynamic metal-coordination bonds (e.g., Fe^3+^ with carboxyl groups) create pH-labile networks: stable at neutral pH, degrading in acid for controlled release [111]. From a release perspective, pH-dependent ionisation increases mesh size and water uptake in the swollen state, thereby lowering diffusional resistance and enhancing drug transport; conversely, partial collapse or reduced ionisation may temporarily restrict diffusion and improve gastric or local protection. Recent examples include pH-responsive chitosan hydrogels designed for drug delivery and gastrointestinal dual-polysaccharide systems based on carboxylated chitosan and carboxymethyl cellulose sodium, in which gastric-to-intestinal pH variation drives sol–gel behaviour, swelling and site-selective drug retention [90,112].

#### 3.1.2. Enzyme-Responsive Systems

These systems are modified by enzymes in the biological microenvironment [113,114,115,116]. Pathological sites may overexpress proteases, esterases or matrix metalloproteinases (MMPs), while the colon contains microbial enzymes capable of degrading selected polysaccharides [61,113,114,117]. Enzymatic cleavage of polymer chains or crosslinks increases permeability, degrades the structure and releases the drug. Reactions include hydrolysis of bonds, destabilisation of colloidal complexes and local structural changes. Enzyme specificity can improve selectivity because release occurs preferentially at enzyme-rich sites such as infected wounds, tumour-associated microenvironments or the colon [61,113,114,115,116,117].

#### 3.1.3. Redox- and Metabolite-Responsive Systems

These respond to internal signals such as redox state, reactive oxygen species (ROS) or metabolites [118,119,120,121,122]. Disulphide bonds (-S-S-) are stable in circulation but can be cleaved in reducing environments, while ROS-sensitive moieties (e.g., thioketal bridges) oxidise in inflammatory environments, altering network properties [118,120,121].

Metabolite-responsive systems use dynamic bonds like boronate esters, which reversibly bind diols (e.g., glucose). This creates closed-loop systems: glucose binding reorganises the network, releasing insulin only during hyperglycaemia [122,123,124]. Response can be tuned at two levels: choice of reactive chemical group, and network parameters (cross-linking density, mesh size) that determine whether the chemical change causes gradual modulation or sudden destabilisation [15]. In hydrocolloid-based formulations, these redox- or metabolite-sensitive motifs are frequently introduced into hybrid architectures rather than arising from the native polysaccharide alone, but the hydrocolloid matrix still governs hydration, retention, and the overall release profile once the trigger has been activated. A recent pectin-based example is an amino-acid-modified HG-type pectin hydrogel in which dynamic cross-linking enables dual pH/ROS-responsive release, illustrating how native polysaccharide chemistry can be combined with pathology-associated oxidative cues [91].

#### 3.1.4. Thermo-Responsive Systems

These utilise temperature-induced phase transitions, often based on polymers with a lower critical solution temperature (LCST), such as PNIPAM-containing or poloxamer-based systems [124,125,126]. Below the LCST, chains are hydrated and soluble; above it, hydrophobic interactions dominate, causing chain collapse, viscosity increase or gelation.

Temperature can be endogenous (body heat triggers in situ gelation of an injected sol) [88,89] or externally applied (NIR or magnetic field heating). Injectable systems that flow at room temperature and gel at 37 °C enable minimally invasive depot formation. The sol–gel transition sharply reduces diffusion, enabling prolonged release [18,124]. This concept is illustrated by recent injectable thermosensitive hydrogel depot systems developed for sustained subcutaneous drug delivery, where body-temperature-triggered gelation creates a local depot and slows release [127].

Biocompatibility is critical: cross-linking density affects not only release but also cell infiltration and hypoxia. Degradation must yield non-toxic, renally clearable fragments [125]. Immunogenicity depends on composition and impurities; PEGylation reduces protein adsorption [126]. Design must synchronise gelation, mechanics, degradation, and immune interactions [5]. In the specific context of hydrocolloid-based systems, thermo-responsiveness often appears in hybrid formulations in which a polysaccharide matrix is combined with thermosensitive synthetic segments or copolymers, while the hydrocolloid component provides mucoadhesion, biocompatibility, injectability, or cargo retention [128]. For this reason, temperature-triggered systems should be interpreted here primarily as hydrocolloid-centred hybrid constructs rather than as a separate class of smart polymers.

#### 3.1.5. Other Physically Triggered Systems

These systems are activated by externally applied energy rather than by a local biochemical change in the microenvironment [16,17,36,129,130,131]. Magnetic hydrogels usually contain iron oxides, ferrites or related particles dispersed in alginate, chitosan, dextran, or hybrid matrices. Under static or alternating magnetic fields, these inclusions can produce magneto-mechanical deformation or magnetothermal heating, which changes mesh size, softens the gel, or accelerates diffusion from the loaded depot [17,129]. Such strategies are especially attractive when clinicians need spatial control after administration, because the trigger can be switched on only over the selected anatomical site.

Light- and ultrasound-responsive systems offer a similar principle of remote control. Photoresponsive chromophores or plasmonic nanostructures convert irradiation—most often in the NIR range—into local heat or bond cleavage, resulting in network relaxation, permeability changes, or rapid release [16,130]. Ultrasound, by contrast, acts through acoustic cavitation and pressure waves that transiently disrupt the hydrogel microstructure and enhance mass transport [36]. For hydrocolloid-based systems, the central design challenge is to couple the external energy absorber to a biocompatible matrix strongly enough to ensure responsiveness, but not so strongly that baseline stability or cargo integrity is lost [131]. In practical terms, these externally triggered systems are rarely “all-hydrocolloid” constructs; rather, they are hydrocolloid-based hybrid platforms in which the matrix ensures hydration, conformability, and biological compatibility, while the embedded responsive component converts external energy into a structural or diffusional change.

#### 3.1.6. Multi-Responsive Systems

These combine two or more stimuli for complex control [132]. Integration of, e.g., pH and temperature, or magnetic field and light, enables “AND” logic gating—drug release only when multiple pathological conditions coincide, minimising off-target activation [35,133].

Advantages include synergistic responses, sequential activation (the first stimulus primes the network, the second triggers release), and resistance to false signals. Examples include hydrogels responding to pH + NIR [134] or magnetic field + temperature [135]. Such systems offer higher functionality and adaptability for medical applications [16,136]. For hydrocolloid-based delivery systems, the main value of multi-responsiveness lies in the possibility of combining a biocompatible, highly hydrated matrix with selective trigger combinations that better match the complexity of real disease microenvironments. However, these systems are also the most structurally complex and, in many cases, depend on hybrid rather than purely natural hydrocolloid architectures [64]. The pH/ROS-responsive pectin hydrogel example also illustrates this multi-responsive logic, because release is controlled by both environmental acidity and oxidative stress rather than by a single trigger [91].

### 3.2. Core Principles for Controlled and Targeted Delivery

Beyond responsiveness, effective design requires mastering the principles that govern transport, stability, and targeting. Design involves compromises [137].

#### 3.2.1. Polymer Network Architecture

Polymer network architecture controls not only whether a hydrocolloid behaves as a depot, adhesive film, injectable sol–gel precursor, or swelling matrix, but also how it responds once exposed to a trigger. Key parameters include mesh size, cross-linking density, segment mobility, charge distribution, and structural heterogeneity, all of which determine equilibrium swelling, mechanical resistance, diffusional permeability, and the extent of stimulus amplification [57,88,89]. A loosely cross-linked network may favour rapid hydration and faster release, whereas a dense or multi-network structure can slow transport and better preserve local retention.

Architecture becomes even more important in hybrid hydrocolloid systems. Nanoparticles, micelles, dynamic coordination domains, or interpenetrating networks can create secondary diffusion barriers, additional drug-binding sites, or mechanically reinforced regions. As a result, the same trigger—such as a pH shift or a temperature increase—may generate very different release profiles depending on whether the network responds by gradual deswelling, pore opening, bond cleavage, or bulk erosion. For this reason, the architecture of the carrier should be treated as a design variable equal in importance to polymer chemistry itself. Recent post-2024 literature has further emphasised that in all-polysaccharide and hybrid PEG–polysaccharide systems, cross-linking strategy and network hierarchy are not secondary formulation details but central determinants of responsiveness, self-healing, and release behaviour [64,128].

#### 3.2.2. Transport Mechanisms and Release Kinetics

Release typically combines Fickian diffusion, matrix relaxation (Case II), erosion, and degradation. Kinetics are described by empirical models (zero-order, Higuchi, Korsmeyer-Peppas), but mechanistic models integrating diffusion with network deformation are increasingly used [63,65]. Flory–Rehner theory helps predict erosion [138]. The specific trigger determines which transport contribution becomes dominant. For example, pH-induced ionisation increases water uptake and enlarges effective mesh size, thereby facilitating diffusion; enzyme-triggered cleavage shifts the mechanism toward matrix erosion or permeability increase; and external physical stimuli often act by transiently lowering diffusional resistance through local softening, pore opening, or microstructural disruption. For this reason, the summary presented in Section 3.2.4 should be interpreted not as a simple list of trigger classes, but as a summary of the structural routes by which responsiveness is translated into measurable release.

#### 3.2.3. Targeting Strategies and Functional Architectures

Targeting strategies in hydrocolloid-based systems operate at several hierarchical levels. Passive targeting exploits naturally occurring gradients such as the enhanced permeability and retention (EPR) effect, local acidosis, enzyme overexpression, oxidative stress, or mucus interactions, thereby enriching the carrier at the desired site without requiring a specific ligand [85]. Active targeting adds a second recognition layer through surface functionalisation with peptides, antibodies, sugars, folic acid, or related ligands that promote receptor-mediated adhesion, uptake, or tissue retention [139,140,141]. Beyond these two classical modes, functional architectures such as core–shell particles, multilayer films, and compartmentalised hydrogels make it possible to spatially separate protection, targeting, and release functions. This is particularly important for polysaccharide-based systems, where one domain may provide mucoadhesion or enzymatic susceptibility while another controls cargo diffusion or ligand presentation. In practice, hydrocolloids contribute to targeting not only as passive matrix materials but also through intrinsic properties such as bioadhesion, mucosal affinity, enzymatic susceptibility, and local tissue conformability, which may enhance retention even before ligand-mediated active targeting is introduced.

#### 3.2.4. Modelling and Design Trade-Offs

Design is multi-criteria: increasing cross-linking density prolongs release but may exclude large drugs; low density accelerates transport, but risks burst release [63,142]. Covalent networks offer stability; dynamic bonds enable stimulus-triggered modulation but require precise modelling. Mechanistic models (Flory–Rehner + transport equations) allow prediction of release profiles at the design stage, reducing empirical optimisation.

Conscious trade-off management between structure, hydration, transport, and degradation is the foundation of rational design. Table 2 provides an overview of the main stimulus-responsive routes relevant to hydrocolloid-based matrices.

**Figure 4:**
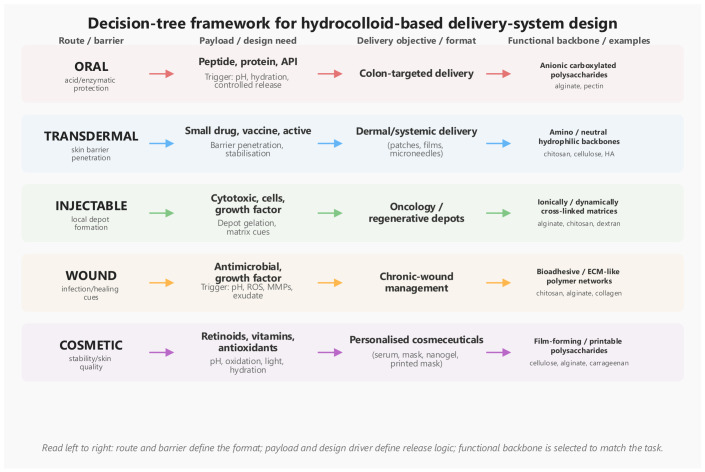
Decision-tree framework for hydrocolloid-based delivery-system design. The scheme links the route of administration or application site with the main biological barrier, payload type, trigger or design driver, intended delivery objective, formulation format, and the corresponding functional hydrocolloid backbone or polymeric group. Representative examples include anionic carboxylated polysaccharides, cationic aminopolysaccharides, neutral hydrophilic backbones, sulfated marine polysaccharides, and ECM-mimetic polymers. The framework emphasises that material selection should be guided by the delivery task, functional chemistry, and the biological barrier rather than by polymer origin alone.

A schematic summary linking material class, trigger logic, and representative application pathways is provided in Figure 4.

## 4. Pharmaceutical Applications

Hydrocolloids contribute to modern drug delivery because their hydration, biocompatibility, bioadhesion, and controlled swelling can be tuned for specific administration routes [2,3]. Initially used as passive excipients, they have evolved towards technologically advanced carriers capable of precisely modulating the release kinetics of therapeutic substances in response to specific physiological conditions [5,15]. Their value, however, depends on matching the polymer network to the biological barrier, the physicochemical properties of the API and the intended release window. In the context of the present review, the most relevant examples are hydrocolloid-rich systems in which alginate, chitosan, hyaluronic acid, pectin, carrageenan, dextran, gellan gum, collagen, or cellulose derivatives determine protection, swelling, retention, or release behaviour. As schematically summarised in Figure 4, these material classes map differently onto oral matrices, transdermal formats, injectable depots, wound dressings, and regenerative constructs, rather than contributing equally across all routes. The following subsections therefore move from broad material classes to more specific formulation examples: alginate/pectin oral beads for IBD-related delivery, chitosan/cellulose gastrointestinal in situ systems, HA- or chitosan-based microneedle patches, injectable thermosensitive depots, fucoidan-based local chemotherapy hydrogels and smart wound patches that combine trigger-responsive therapy with monitoring [30,112,127,144,146,147].

For this reason, the most informative case studies are those that connect four elements: polymer composition, API structure, interaction with the administration medium and measured release behaviour. In oral systems, ionisation, mucus interaction and enzymatic degradation usually dominate; in transdermal and cosmetic systems, hydration, skin residence, partitioning and barrier modulation are more important; in injectable depots, gelation kinetics, modulus, erosion, and local tolerability become decisive. This route-specific logic is used below to avoid treating all hydrocolloids as interchangeable carriers.

### 4.1. Oral Delivery Using Smart Hydrocolloid Systems

The oral route of administration is the most preferred by patients, yet it poses an enormous challenge for molecules sensitive to the acidic environment of the stomach or those poorly soluble in water. Hydrocolloid systems offer effective solutions to these problems through the physicochemical modification of carriers [5,148].

In oral delivery, the most useful hydrocolloid case studies should be read through medium-polymer-API coupling. Acidic alginate or pectin networks can contract under gastric conditions and then swell or erode at higher intestinal pH; cationic chitosan adds mucoadhesion and epithelial interaction; dextran, locust bean gum and related polysaccharides rely more strongly on microbial degradation. For hydrophilic peptides, the main task is acid/protease protection followed by release after transit. For hydrophobic small molecules, the main task is dispersion, solubilisation and prevention of aggregation inside a hydrated carrier. These are different design problems and should not be presented as one generic oral-hydrogel mechanism.

#### 4.1.1. Improving Bioavailability of Poorly Water-Soluble Drugs

Many modern active pharmaceutical ingredients (APIs) exhibit a highly hydrophobic nature, which drastically limits their absorption. The use of hydrogels with incorporated nanostructures (e.g., micelles, cellulose nanocrystals) allows for an increase in the specific surface area of the drug and an improvement in its solubility. Hydrocolloids, such as modified cellulose or hyaluronic acid, act as stabilisers, preventing the aggregation of hydrophobic molecules in the gastrointestinal tract [57,149].

For hydrophobic APIs, the hydrocolloid usually improves apparent bioavailability indirectly rather than by dissolving the drug itself. Its functions are to stabilise nanostructured domains, limit aggregation during gastrointestinal dilution, maintain a hydrated diffusion pathway and control residence time. The limitation is clear: highly swollen polysaccharide networks often have low affinity for non-polar drugs unless a secondary carrier, lipid phase, micelle, surfactant or other hydrophobic domain is introduced.

#### 4.1.2. Protection of Peptides and Proteins

Biologic drugs, including insulin and other peptide hormones, undergo rapid denaturation under the influence of low stomach pH and proteolytic enzymes (e.g., pepsin). The use of pH-responsive hydrogels, e.g., based on sodium alginate or DNA-motifs, allows for the physical shrinkage of the matrix in an acidic environment, which effectively hides and protects the sensitive protein [138,150]. Only after passing into the intestines, where the pH rises, does the network undergo deprotonation and rapid swelling, releasing the intact drug [20,43,151].

The key interaction in peptide and protein delivery is a pH-controlled protection-release sequence. A compact, protonated or ionically cross-linked network can slow access of acid and proteases in the stomach, while deprotonation or ion exchange in intestinal fluid increases charge repulsion, osmotic pressure and mesh size. This mechanism is useful only if the protective barrier does not cause irreversible adsorption, denaturation or a large burst immediately after swelling.

#### 4.1.3. Controlled and Prolonged Drug Release

Hydrophilic matrices, particularly those based on cellulose derivatives (e.g., HPMC, CMC), are commonly used to create prolonged-release tablets. Upon contact with gastrointestinal fluids, the polymer swells, forming a gel barrier layer that slows down drug diffusion and the erosion of the tablet itself, enabling release kinetics approaching zero-order [58,65].

#### 4.1.4. Site-Specific and Colon-Targeted Delivery

In the treatment of inflammatory bowel disease (IBD), delivering the drug directly to the colon is crucial. Plant and microbial polysaccharides (e.g., guar gum, dextran, locust bean gum (LBG)) are utilised for this purpose, as they are completely resistant to digestion in the upper sections of the gastrointestinal tract [13,152]. Their degradation only occurs in the large intestine under the influence of specific bacterial flora producing appropriate enzymes (e.g., azoreductases, glycosidases), which release the drug directly at the site of inflammation [61,117]. Recent overviews of oral colon-specific systems further confirm that polysaccharides such as chitosan, pectin, alginate, dextran, and guar-based carriers remain among the most attractive platforms because they combine upper-GIT protection with microflora-triggered or pH-assisted release in the colon [43].

For colon-targeted systems, the polymer has to remain sufficiently intact during gastric and small-intestinal transit but become susceptible to microbial enzymes or local pH conditions in the colon. Drug structure matters here: weak acids and bases may change ionisation and partitioning along the gastrointestinal tract, whereas large or hydrophilic molecules depend more strongly on mesh size, erosion and enzymatic breakdown.

#### 4.1.5. Mucoadhesive Systems and Absorption Enhancement

Polymers with mucoadhesive properties, particularly cationic chitosan, can interact electrostatically with the negatively charged mucus layer (mucin) lining the intestines. This causes a significant prolongation of the residence time of the drug formulation at the absorption site [44]. Additionally, chitosan exhibits the ability to transiently open tight junctions between epithelial cells, significantly increasing the paracellular transport of macromolecules [153].

### 4.2. Transdermal Drug Delivery Systems

Administering drugs through the skin avoids the first-pass effect through the liver and degradation in the gastrointestinal tract, ensuring a constant therapeutic concentration in the plasma. Hydrogels constitute a flexible, biocompatible transfer platform here [154].

Transdermal examples should therefore distinguish local dermal deposition from true systemic delivery. Hydrophilic hydrocolloids mainly hydrate and soften the skin surface, whereas microneedles, permeation enhancers, iontophoresis or nanocarriers are usually required when the payload is large, charged or poorly partitioning into the stratum corneum.

#### 4.2.1. The Stratum Corneum Barrier and Its Modulation

The main obstacle in transdermal administration is the stratum corneum. Hydrogels, thanks to their high water content, act as strong hydration promoters, leading to the loosening of the intercellular lipid packing of the epidermis and facilitating the penetration of active substances [68,155].

#### 4.2.2. Controlled and Sustained Transdermal Release

Hydrocolloid-based transdermal systems enable precise control of the API diffusion rate from the patch to the skin. Optimising the polymer’s cross-linking density allows for the creation of matrices that release painkillers (e.g., diclofenac) or hormones uniformly over many hours or days [92,156].

#### 4.2.3. Microneedle-Integrated Hydrogel Systems

Microneedle systems have expanded the transdermal delivery options for macromolecules (vaccines, insulin) by painlessly piercing the stratum corneum without stimulating pain receptors [157]. Dissolvable microneedles are often made from biopolymers such as hyaluronic acid, which rapidly degrade in the skin’s interstitial fluid after application, releasing a precisely loaded dose of the drug directly into the capillaries [68,158]. Recent post-2024 work continues to support hyaluronic-acid-based dissolving or hydrogel-forming microneedles as one of the most practical hydrocolloid-centred strategies for minimally invasive transdermal delivery, particularly where local dermal deposition and controlled dissolution are both required [87].

From a material-design perspective, hyaluronic-acid-based microneedles are attractive because the same polymer can provide mechanical insertion when dry and rapid hydration or dissolution after insertion. The trade-off is direct: increasing molecular weight or cross-linking may improve strength and handling, but can slow dissolution and reduce dose release; lowering network density improves release but may compromise insertion and shelf stability.

#### 4.2.4. Stimuli-Responsive Transdermal Hydrogels

Innovative wearable hydrogel patches can release drugs in response to external or internal stimuli. For example, systems based on thermoresponsive poloxamers can increase the release of substances as body temperature rises with inflammation [159,160]. Furthermore, matrices integrated with miniature iontophoresis systems actively drive the drug deep into the tissues using a weak electrical current [161].

#### 4.2.5. Localised and Systemic Drug Delivery

The same transdermal hydrogel platform can be tuned either for local therapy or for systemic administration, but the design priorities differ substantially. For local dermatological use, the objective is usually to maximise retention within the epidermis or superficial dermis while minimising deeper permeation; this is desirable for anti-inflammatory, antimicrobial, anti-psoriatic, or antineoplastic agents intended to act at the site of skin pathology. By contrast, systemic delivery requires steady flux across the full skin barrier and a sufficiently large drug reservoir to maintain therapeutic plasma levels over time. Hydrocolloids enable both strategies because their water content, adhesiveness, and cross-linking density can be adjusted together with the use of microneedles, permeation enhancers, or electrical assistance. Hydrocolloid choice strongly affects this balance: HA- and chitosan-rich microneedle or patch systems often favour local retention and skin compatibility, whereas more reservoir-like cellulose-based matrices are more often developed for sustained systemic flux. In practice, the distinction between local and systemic delivery is not binary but exists on a continuum defined by drug potency, target depth, and acceptable systemic exposure [87,162].

This distinction is also important for cosmetic and cosmeceutical claims. A formulation that increases hydration or superficial skin deposition should not be described as a systemic transdermal delivery system unless flux, target depth and exposure are demonstrated.

### 4.3. Injectable Depot Formulations

Injectable in situ forming hydrogels combine the advantages of minimally invasive administration via a syringe needle (as a liquid sol) with the capability of immediate gelation at the target site (at 37 °C), forming a stable drug depot [17,18,103].

In injectable depots, the strongest examples are those in which sol–gel transition, modulus and erosion are matched to the intended therapeutic window. A hydrocolloid-rich depot must be injectable through a practical needle, gel quickly enough to avoid leakage, retain the API locally and degrade without harmful residues. These requirements often conflict, so depot design is a compromise rather than a single optimisation problem.

#### 4.3.1. Long-Acting Delivery of Small-Molecule Drugs

Injectable depot systems are being explored as tools for longer-acting local or systemic therapy in chronic and oncological diseases [109]. The injection of a gelling implant releases the drug slowly, in line with the progressive erosion of the matrix. In selected local-delivery settings, this approach may reduce administration frequency or systemic exposure, but the effect depends on the drug, indication, dose, depot residence time and release profile [17]. Recent thermosensitive injectable depots for sustained subcutaneous release illustrate the same principle: after administration, gelation converts a low-viscosity formulation into a local drug reservoir [127].

Small molecules introduce an additional partitioning problem. Hydrophilic drugs may diffuse too rapidly from a swollen network and cause burst release, whereas hydrophobic drugs may require micelles, nanoparticles or hydrophobic domains for adequate loading. A convincing case study should therefore report not only release duration but also loading efficiency, burst fraction, depot integrity and local tolerability.

#### 4.3.2. Injectable Depots for Peptides and Protein Therapeutics

Proteins and growth factors are highly susceptible to rapid in vivo degradation. Injectable hydrocolloid networks shield these macromolecules, maintaining their bioactivity [163]. These systems allow for the controlled release of, e.g., vascular endothelial growth factor (VEGF) directly into the ischaemic heart muscle or damaged tissues, promoting neovascularisation [164,165].

For localised anticancer therapy, the matrix should be interpreted as a retention and exposure-control tool rather than as a universal substitute for systemic treatment. The hydrocolloid can reduce washout from the resection cavity or injection site, but selectivity still depends on the payload, local pH/enzyme/ROS conditions, release gradient and tissue penetration depth.

#### 4.3.3. Localised Therapy and Site-Specific Drug Retention

Hydrogels injected directly into postoperative cavities (e.g., after brain glioblastoma resection) prevent tumour recurrence. The use of responsive DNA or chitosan hydrogels allows the carrier to immediately halt postoperative tissue haemorrhage, and then slowly destroy surviving cancer cells by releasing cytotoxins exclusively in the acidic residual microenvironment [81,166]. Fucoidan hydrogel systems proposed for hepatocellular carcinoma provide another example of local chemotherapy, with the polysaccharide contributing both to the matrix structure and to the localised therapeutic concept [30].

#### 4.3.4. Injectable Hydrogels in Regenerative Therapies

In regenerative medicine, injectable hydrogels are valuable not merely as passive cell carriers but as instructive microenvironments. Their shear-thinning or in situ gelling behaviour protects cells during injection, while the hydrated network subsequently supports nutrient transport, cell spreading, and local retention inside irregular defects. For MSC delivery in cartilage, bone, or soft tissue repair, this means that the hydrogel can simultaneously improve post-administration cell survival, shield the cells from washout, and provide biochemical or mechanical cues closer to the native ECM than a simple suspension. In this setting, injectable alginate, hyaluronic acid, chitosan, dextran, or gellan-based depots are particularly attractive because they can be formulated as shear-thinning, ionically cross-linked, or in situ gelling systems that improve retention inside irregular defects while simultaneously serving as carriers for cells, cytokines, or mineral phases [162]. When loaded additionally with growth factors or mineral phases, the same depot can coordinate sequential cell delivery and tissue remodelling, making injectable hydrocolloid systems particularly attractive for minimally invasive regenerative therapies [165,167,168].

### 4.4. Hydrocolloids for Wound Care and Tissue Repair

Polymer dressings have increasingly complemented, and in some indications replaced, traditional passive gauzes in treating difficult and chronic wounds, such as diabetic foot ulcers or burns, offering active management of the healing process [169,170].

In wound applications, hydrocolloids are most persuasive when moisture handling, mechanical protection, antimicrobial action and bioactive release are linked to the wound microenvironment. Chronic wounds are chemically heterogeneous: pH, exudate volume, protease activity, oxidative stress and microbial burden can vary strongly between patients and healing stages. This variability explains why a responsive dressing should be tested under more than one simplified buffer condition.

#### 4.4.1. Moisture Management and Microenvironment Regulation

A key tenet of wound care is maintaining optimal moisture [92]. Hydrogels exhibit a unique, dual capability: they can absorb excess wound exudate (preventing the maceration of healthy edges) while simultaneously donating moisture to dry and necrotic wounds, which accelerates autolytic debridement and keratinocyte migration [86].

#### 4.4.2. Antimicrobial and Anti-Inflammatory Hydrocolloid Systems

Chronic wounds are heavily burdened not only by resistant biofilms but also by a protease-rich inflammatory microenvironment that prevents orderly tissue repair. Advanced hydrocolloid matrices therefore aim to do more than simply kill planktonic bacteria. Cationic chitosan can disrupt bacterial membranes, sequester negatively charged cell-wall components, and interfere with biofilm organisation, while silver, zinc oxide, iodine, or antibiotic-loaded particles broaden the antimicrobial spectrum and provide prolonged local action [45,46,131]. Recent reviews of natural polysaccharide wound dressings likewise identify alginate, chitosan, dextran, hyaluronic acid, and composite polysaccharide systems as especially relevant because they combine haemostatic or antimicrobial effects with exudate control and maintenance of a hydrated wound environment [171]. At the same time, the hydrogel matrix can dilute inflammatory mediators, absorb excess exudate, and reduce repeated mechanical trauma during dressing changes. The most effective systems combine these functions so that antimicrobial action, moisture management, and inflammation control occur together rather than as separate treatment steps. This concept is also relevant to natural antimicrobial compositions, including essential-oil-based systems, where carrier composition and physicochemical dispersion parameters can influence biological activity [171,172].

Mechanistically, antimicrobial hydrocolloid dressings operate through several layers: the matrix controls exudate uptake and contact time; cationic groups can interact with bacterial membranes and biofilms; and loaded inorganic or antibiotic components provide additional antimicrobial action. The limitation is that stronger antimicrobial loading can also increase cytotoxicity or delay re-epithelialisation, so antimicrobial efficacy should be balanced against fibroblast and keratinocyte compatibility.

#### 4.4.3. Bioactive and Regenerative Hydrogel Dressings

Modern hydrogel dressings also serve as regenerative microenvironments that actively instruct the healing cascade. By releasing growth factors, peptides, nucleic acids, or extracellular-matrix-derived cues, they can recruit fibroblasts, support keratinocyte migration, and stimulate angiogenesis within the wound bed [51]. Polysaccharide-based regenerative dressings are increasingly designed as composite matrices that release growth factors, peptides, nucleic acids, or cell-derived signals while simultaneously supporting angiogenesis, fibroblast activity, and macrophage polarisation [171]. Equally important is immunomodulation: a well-designed hydrocolloid matrix can shift macrophage behaviour away from persistent M1-dominant inflammation toward an M2-like reparative phenotype, thereby promoting matrix deposition, vascularisation, and re-epithelialisation [171,173].

This regenerative concept is especially relevant for diabetic and other chronic wounds, in which healing fails not because of a single deficit but because inflammation, infection, poor vascularisation, and defective matrix remodelling coexist. Hydrocolloid dressings that combine structural support with bioactive release are therefore closer to temporary therapeutic tissues than to conventional passive coverings.

#### 4.4.4. Smart and Responsive Wound Dressings

A further level of functionality is offered by patches that respond to local pathologies [19]. These dressings detect the overexpression of specific enzymes (e.g., matrix metalloproteinases (MMPs)), the alkaline pH of an infected wound, or high levels of reactive oxygen species (ROS). In response to this stimulus, the gel structure loosens, releasing an optimal dose of an antibacterial agent proportional to the severity of the infection [116]. Recent theranostic microneedle platforms take this idea further by combining NIR-triggered antibacterial action with real-time wound pH monitoring, so that diagnosis, external activation and treatment are integrated in one patch format [146].

Responsive wound patches should also be treated cautiously. A pH-, enzyme-, or ROS-triggered release profile is useful only if the trigger window corresponds to the pathological range in real wound fluid and if the released dose remains within a safe local concentration range.

### 4.5. Translational Progress and Clinical Perspectives

The transition of smart hydrocolloid systems from the laboratory phase to widespread clinical use (bench-to-bedside) involves specific technological and regulatory challenges [101]. Recent overviews also emphasise that formulation complexity, nanocomponent integration, and hybrid architectures may improve performance, but they simultaneously intensify regulatory, manufacturing, and scalability burdens [165].

For translation, successful hydrocolloid products should be framed around functions that are robust, reproducible and familiar to regulators: barrier protection, moisture management, viscoelastic filling, lubrication and local retention. Highly responsive closed-loop systems remain more developmental. This distinction explains why commercial uptake is stronger for conventional hydrocolloid dressings and hyaluronic-acid fillers than for autonomous smart patches.

**Figure 5:**
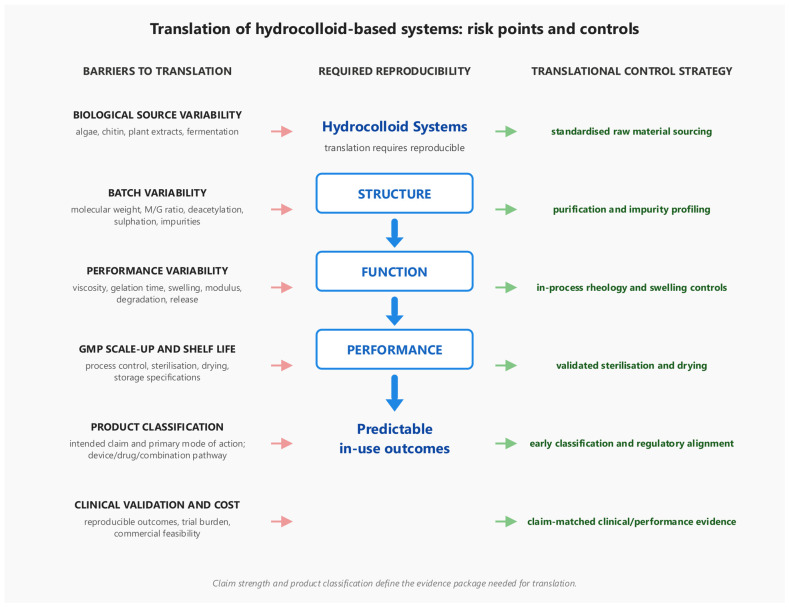
Interconnected barriers limiting the clinical and commercial translation of hydrocolloid-based systems. The arrows indicate dominant relationships rather than exclusive one-to-one dependencies. Variability in biological source and batch composition affects structural, functional, and performance reproducibility, which in turn determines the predictability of in vivo outcomes. Translational control therefore requires standardised sourcing, impurity profiling, in-process rheological and swelling controls, validated sterilisation and drying, early regulatory alignment, and claim-matched clinical evidence.

Interconnected barriers limiting clinical and commercial transition of hydrocolloid-based systems are summarised in Figure 5.

The barriers in Figure 5 overlap in practice. For naturally derived hydrocolloids, batch variability is not a minor raw-material issue; differences in molecular weight, degree of deacetylation, alginate M/G ratio, sulphation pattern of marine polysaccharides, residual protein or endotoxin content, and extraction history can affect viscosity, gelation time, swelling ratio, degradation rate, and elastic modulus. Such shifts may alter release kinetics or adhesive performance, and during scale-up they often require adjustments to polymer concentration, cross-linker level, sterilisation conditions, or the drying process. The same variability then affects GMP reproducibility and shelf-life specifications. For responsive or combination systems, the regulatory burden is greater because the product has to be evaluated not only as a material but also as a delivery platform whose performance depends on trigger sensitivity, biological safety, active-substance stability and clinical outcome.

#### 4.5.1. Barriers in Clinical Translation

The fundamental problem remains production scalability (up-scaling). Due to the natural origin of hydrocolloids (algae, animal chitin, plant extracts), these polymers exhibit significant batch-to-batch variability. This instability in molecular parameters forces continuous recalibration in industrial reactors to guarantee reproducible drug release kinetics within GMP standards [174].

#### 4.5.2. Regulatory Considerations and Safety Assessment

Complex, responsive hydrogel matrices fall into the legislative grey area of “combination products”. Because they serve a dual function (a physical medical device scaffold combined with the release of an active pharmaceutical ingredient), they are subject to dual and highly stringent safety assessment criteria by the FDA and EMA [102]. This can increase development costs and lengthen approval timelines [103,175,176].

#### 4.5.3. Commercially Available Hydrocolloid-Based Products

Currently, the clinical market is dominated by hydrocolloids serving barrier and structural functions [6]. These primarily include classic hydrocolloid burn dressings, hyaluronic acid used as a viscoelastic dermal filler in aesthetic medicine, and ophthalmic gels preventing dry eye [177]. These examples underline that commercially successful hydrocolloid products still rely mainly on barrier, viscoelastic, or structural functions rather than fully closed-loop responsiveness. Responsive “smart” systems still face substantial translational barriers before routine commercialisation.

#### 4.5.4. Emerging Trends and Future Clinical Directions

Future pharmaceutical development is likely to make increasing use of personalised therapeutic strategies. 3D bioprinting already allows for the creation of multi-compartmental hydrogels tailored more closely to the anatomy of a specific patient (e.g., irregular tissue defects) [96,104]. Moreover, the integration of bioelectronics is leading to the development of sensor-integrated smart patches, where built-in microprocessors continuously monitor wound parameters (pH, glucose levels) and automatically dose the drug in a closed-loop theranostics feedback system [100,165].

## 5. Cosmetic and Cosmeceutical Applications

The conceptual convergence of hydrocolloid-based innovations in pharmaceutical and cosmetic applications is already foreshadowed in Figure 1. Here, however, the overlap is discussed in functional rather than purely chronological terms, with emphasis on how the same material platform may stabilise actives, regulate dermal residence, and support controlled topical delivery.

The current transition towards “smart hydrocolloid systems” should therefore be understood as an extension of classical formulation science rather than its replacement. The same matrix that acts as a thickener or film-former can also protect labile molecules, prolong residence on the skin, modulate dermal penetration, or respond to environmental cues such as pH, temperature, or hydration state. Consequently, hydrocolloids in cosmeceutical design are not merely passive excipients but functional carriers that shape both product performance and the local bioavailability of actives [178].

In contrast to pharmaceutical formulations, cosmetic and cosmeceutical systems are usually intended for topical action with limited or no systemic absorption. Their design therefore prioritises long-term skin compatibility, aesthetics, and formulation stability over systemic pharmacokinetics. Representative hydrocolloid classes, bioactive ingredients, dosage formats, and cosmetic functions discussed in this section are summarised in Table 3.

This distinction should also guide the strength of the claims. In cosmetic and cosmeceutical studies, the evidence base is often formulation-centred and short-term, relying on stability tests, release studies, skin deposition, TEWL, hydration or sensory outcomes rather than long-term clinical endpoints. Therefore, hydrocolloid systems should be described as improving stability, residence time, hydration or local availability unless therapeutic efficacy has been demonstrated under appropriate conditions.

In this section, hydrocolloids are discussed through formulation functions rather than as passive excipients. Retinoids are used as examples of photochemical stabilisation and dermal retention; vitamin C and polyphenols as examples of oxidation-sensitive actives; curcumin and gamma-oryzanol as examples of nanocarrier-assisted hydrogel deposition; HA- and alginate/carrageenan-based masks as examples of hydration and barrier support; and 3D-printed hydrogel masks as examples of personalisation through spatially controlled composition and geometry.

### 5.1. Stabilisation of Labile Cosmetic Actives

The stability of cosmetic active ingredients is frequently limited by photodegradation, oxidation, hydrolysis, and poor chemical compatibility with the formulation environment. Hydrocolloid-based systems address these limitations by creating protective microenvironments that reduce exposure to reactive species, limit oxygen and light diffusion, and modulate local pH and water activity. As a result, stabilisation is inherently coupled with controlled release and improved functional availability of the active compound.

A useful way to read these examples is to separate three functions: chemical protection of the active during storage, controlled release after application, and retention within the superficial skin layers. The same hydrocolloid network may contribute to all three, but the dominant mechanism depends on the active molecule. Hydrophobic and photolabile actives usually require a secondary dispersed phase, whereas hydrophilic and oxidation-sensitive actives are more strongly affected by water activity, oxygen diffusion and local pH.

Related stabilisation challenges also concern volatile natural compositions such as essential-oil-based systems, for which the carrier medium and dispersion properties may affect both physicochemical behaviour and antimicrobial performance [172].

Volatile botanical or essential-oil-based compositions should remain a supporting example rather than a separate theme. They are relevant here only when the hydrocolloid phase controls dispersion, residence time or release at the skin interface; otherwise they would broaden the review beyond hydrocolloid-based delivery systems.

Retinoids provide one of the clearest examples of cosmetic actives that benefit from hydrocolloid-assisted stabilisation. Retinyl palmitate, widely used in anti-ageing formulations, undergoes rapid photochemical degradation in conventional emulsions. Encapsulation in nanoemulsion-hydrogel systems improves stability during storage and under UV exposure, while chitosan-based nanocapsules can additionally enhance dermal retention, illustrating how stabilisation and delivery can be coupled within a single carrier design [10,181,188,189].

For retinoids, the molecular-design issue is not simply encapsulation but compatibility between a hydrophobic, photolabile active and a mostly hydrated carrier. Chitosan shells, nanoemulsion droplets or lipid domains can provide the hydrophobic microenvironment, while the hydrocolloid gel controls viscosity, skin contact and release. Without such a secondary domain, a polysaccharide hydrogel alone may have limited capacity to solubilise or stabilise retinoids.

Recent work also indicates that hydrogel architectures can stabilise more labile retinoids such as retinol. Cross-linked polymer networks, including systems incorporating hyaluronic acid or inorganic fillers, appear to limit oxygen access and molecular mobility within the matrix, thereby improving physicochemical stability. Comparable strategies have also been reported for lutein/retinol co-loaded pH-responsive hydrogel beads, where encapsulation protects the actives while preserving the possibility of controlled release [10,84,189,190].

Hydrocolloid systems are likewise relevant for oxidation-prone antioxidants. Ascorbic acid, one of the least stable cosmetic actives, shows improved preservation when immobilised in PEG-, PVA-, or hydrogel-based matrices that restrict oxygen diffusion and reduce local molecular mobility [191,192]. Chitosan-derived carriers have also been investigated as protective systems for vitamin C and related antioxidants, again linking storage stability with more controlled exposure at the skin interface [54].

For ascorbic acid and related antioxidants, water-rich hydrogels are a double-edged platform. They may reduce oxygen diffusion and molecular mobility in a structured network, but they can also accelerate hydrolysis or oxidation if pH, oxygen exposure, metal impurities and packaging are not controlled. This should be treated as a formulation constraint rather than an automatic advantage of hydrocolloids.

Similar strategies have been reported for polyphenols and flavonoids such as rutin and curcumin. Chitosan-based films and composite polysaccharide hydrogels can protect these molecules against degradation while simultaneously moderating their release profile [182,193]. In more advanced formulations, hydrogel matrices are combined with nanocarriers, for example lipid nanoparticles or micellar systems, which is particularly advantageous for poorly soluble actives such as curcumin or gamma-oryzanol [10,186,187,189].

An important recent direction is the development of hybrid, multi-component platforms in which the hydrocolloid matrix acts not simply as a passive barrier but as an active stabilising environment. Composite hydrogels, hydrogel microspheres, and nanoemulsion-loaded gels can maintain actives in a dispersed state, reduce aggregation or crystallisation, and preserve controlled-release behaviour during storage and application [187,194].

### 5.2. Controlled and Enhanced Dermal Delivery

Hydrocolloid systems enable controlled and enhanced dermal delivery by modulating the release kinetics, retention, and penetration of active ingredients within the skin. Unlike conventional formulations, hydrogel matrices act as reservoirs that maintain prolonged contact with the skin surface, regulate diffusion through their network structure, and modify the microenvironment at the skin interface, including hydration and local concentration gradients.

For clarity, dermal delivery should be divided into release from the formulation, deposition in the stratum corneum or viable epidermis, and true permeation across the skin. Hydrocolloids can improve the first two processes through hydration, adhesion and reservoir effects, but systemic delivery usually requires additional technologies such as microneedles, permeation enhancers, iontophoresis or nanocarriers.

pH-responsive and nanostructured hydrogels provide a representative example of this approach. When active compounds are first encapsulated in nanoparticles and then immobilised within a hydrogel matrix, release becomes environment-dependent, and skin retention is often higher than in non-encapsulated controls. This behaviour has been demonstrated for lutein/retinol-loaded hydrogel beads and for chitosan-based systems, where bioadhesion and electrostatic interactions enhance retention within the stratum corneum and facilitate transport into deeper epidermal layers [181,190].

Hybrid hydrogel-nanocarrier systems further improve dermal delivery by coupling controlled release with enhanced penetration. Liposomal hydrogels containing adapalene, for example, increase skin deposition relative to conventional gels, with the hydrogel acting as a reservoir and the liposomes assisting transport across the lipid barrier [195]. A comparable design logic can also be applied to hydrophilic or unstable actives such as vitamin C, for which the hydrogel phase prolongs residence and attenuates burst release.

Curcumin-loaded hydrocolloid systems illustrate the close link between stabilisation and dermal delivery. Hyaluronic-acid-based micelles or nanogels improve the aqueous stability of this hydrophobic compound and enable controlled release, while in vitro, ex vivo, and in vivo studies indicate enhanced deposition in epidermal layers and improved anti-inflammatory performance compared with free curcumin [186,196]. Comparable hybrid strategies have also been explored for gamma-oryzanol and other lipophilic actives integrated into nanogel systems with photoprotective functions [187].

Curcumin and gamma-oryzanol also illustrate the main limitation of hydrophilic matrices for lipophilic actives: the hydrocolloid is rarely the only carrier. Its role is usually to immobilise micelles, nanogels, lipid particles or other dispersed domains and to regulate their contact with the skin. The critical parameters are therefore not only release rate but also particle stability, active crystallisation, skin deposition and irritation potential.

An important direction in this field is the development of multifunctional matrices that integrate delivery with additional protective roles, such as photoprotection, barrier support, or irritation control. In these systems, the hydrocolloid network does not merely carry the active ingredient; it also determines residence time, local distribution, and the balance between superficial retention and deeper permeation [179,187].

Hydrocolloids may additionally contribute to skin conditioning through their own physicochemical or biological properties. Hydration, film formation, bioadhesion, and extracellular-matrix-mimetic behaviour can all enhance the perceived and functional performance of the formulation independently of the encapsulated active, further blurring the distinction between carrier and bioactive component [9,179].

### 5.3. Bioresponsive Skincare: Moisture Regulation and Barrier Support

Hydrocolloids are widely used in skincare because their water-binding capacity and hydrophilic network structure allow them to modify the microenvironment of the stratum corneum. By absorbing and retaining water, hydrogel matrices can increase surface hydration and reduce transepidermal water loss (TEWL), while their semi-occlusive character helps preserve comfort without fully blocking gas exchange [179,197,198].

The moisturising effect should be interpreted as a function of water binding, film formation and semi-occlusion rather than as proof of biological repair. TEWL and hydration improvements can be transient and depend on polymer molecular weight, humectants, film integrity, application time, ambient humidity and the condition of the skin barrier.

Hyaluronic-acid-based hydrogels are among the most intensively studied moisturising materials. Because HA binds water in amounts far exceeding its own mass, topical HA gels and masks can produce measurable increases in skin hydration and, in many studies, concomitant reductions in TEWL [179]. Related barrier-support functions have also been described for marine polysaccharides such as alginate and fucoidan, whose film-forming and extracellular-matrix-associated properties contribute to water retention at the skin surface [9,50].

The hydration effect of hydrogels is closely connected to the barrier they create on the skin. In vivo TEWL measurements show that well-designed hydrogel films can reduce water loss while maintaining a degree of breathability, which is important for comfort and for prolonged cosmetic use [197,198].

Polysaccharide matrices can also work synergistically with bioactive ingredients. For example, carboxymethylated chitosan combined with plant extracts has been reported to improve epidermal moisture while simultaneously supporting antioxidant and antimicrobial functions, illustrating how moisturisation and active delivery can be integrated within a single formulation [183]. Clinical and translational studies likewise suggest that hydrocolloid-containing moisturisers can improve barrier-associated parameters when combined with suitable functional additives [50,199].

A broader implication of these findings is that hydrocolloid films and masks should not be viewed only as passive humectant layers. Their internal architecture governs how water is stored, transferred, and released at the skin surface, and therefore determines whether the formulation acts as a short-lived wetting agent or as a sustained barrier-support system [179,198].

Contemporary bioresponsive skincare thus draws conceptually from medical hydrogel technologies, particularly from wound-dressing research, where adaptive swelling and controlled hydration are used to stabilise the local microenvironment. Transferred to cosmetology, the same principles support sustained moisturisation, reduced application frequency, and more functionally designed barrier-support products [18,179,198].

### 5.4. Functional Convergence Between Cosmetic and Pharmaceutical Hydrocolloid Systems

The previous subchapters show that modern hydrocolloid systems used in cosmetics increasingly rely on design principles well established in pharmaceutical sciences, including controlled release, protection of labile compounds, and responsiveness to environmental stimuli. As a result, the technologies used in cosmetics and pharmaceuticals are becoming increasingly similar, although the regulatory boundary remains strict and claim-dependent. Hydrocolloids are no longer used only as rheology modifiers or stabilisers but are increasingly engineered as active systems that modulate the local bioavailability and performance of cosmetic ingredients.

This convergence should be framed technologically, not regulatorily. Cosmetic and pharmaceutical formulations may use similar carriers, release tests, and skin models, but their permitted claims, evidence requirements, and risk-benefit logic remain different. This distinction is particularly important for injectable HA products, microneedle formats, and formulations described with terms such as regenerative, therapeutic or bioactive.

A key manifestation of this convergence is the transfer of drug-delivery strategies into cosmetic formulations. Encapsulation in nanocarriers, incorporation into responsive hydrogel matrices, and control over release kinetics are increasingly used to improve the efficacy, tolerability, and predictability of retinoids, antioxidants, vitamins, and other cosmetic actives. In this sense, hydrogel-based platforms provide a common technological framework for stabilisation, delivery, and controlled interaction with biological barriers in both pharmaceutical and cosmetic contexts [179,200].

This overlap becomes especially clear in aesthetic medicine, where injectable hyaluronic-acid-based hydrogels combine mechanical support, hydration, and bioactive signalling functions. Such products sit at the interface between dermal therapeutics, regenerative medicine, and advanced cosmetic intervention, and they are therefore better understood as translational hydrogel systems than as conventional passive cosmetics [52,180].

Skin-booster formulations illustrate this intermediate space particularly well. These lightly cross-linked, or low-viscosity hyaluronic-acid systems are designed not for volumetric augmentation but for improving hydration, elasticity, and overall skin quality after intradermal administration. Their development shows how cosmetic objectives are increasingly pursued using formulation principles borrowed from injectable therapeutics, including rheological tuning, controlled tissue distribution, and prolonged local residence [52,180].

Methodological convergence is equally important. In vitro release testing, ex vivo skin permeation models, and advanced imaging of skin distribution—once mostly associated with pharmaceutical development—are now routinely used in cosmeceutical research. This shift enables a more quantitative relationship between formulation design and biological outcome, moving cosmetic science closer to an evidence-based framework [179].

Overall, hydrocolloid-based systems exemplify a broader transformation in cosmetic science, where materials originally developed for drug delivery are adapted to create multifunctional formulations with both protective and bioactive roles. This evolution underpins the growing category of cosmeceuticals, in which hydrogel matrices act not only as carriers but also as active interfaces that dynamically interact with the skin [179,200].

### 5.5. Personalisation and Advanced Manufacturing: The Case of 3D-Printed Masks

The evolution of hydrocolloid systems increasingly incorporates advanced manufacturing technologies, enabling a shift from standardised formulations toward highly personalised cosmetic products. Among these, extrusion-based 3D printing techniques, adapted from pharmaceutical and biomedical applications, allow the precise spatial deposition of hydrogel materials with controlled composition and architecture. This approach enables not only the fabrication of complex geometries but also the localisation of active ingredients within defined regions of the final product.

In cosmetic applications, 3D printing has already been explored for personalised hydrogel-based facial systems. Extrusion-printed patches or masks based on iota-carrageenan and sodium hyaluronate can be tailored to specific skin types or functional goals by varying humectants, botanical extracts, or other additives, demonstrating the feasibility of personalised hydrocolloid-based cosmetics [11,184].

At present, 3D-printed hydrocolloid masks should be presented as an emerging manufacturing concept rather than as a mature clinical or commercial standard. The key unresolved issues are microbial control, reproducible rheology, drying or packaging stability, dose uniformity across printed regions, consumer-specific design validation and evidence that spatially programmed composition improves performance beyond conventional masks.

From a materials perspective, hydrocolloids are especially suitable for such applications because their rheology can be tuned to achieve shear thinning, shape retention, and post-deposition gelation. More broadly, work on hydrogel bioinks shows that balancing viscosity, cross-linking density, and mechanical stability is essential if printability is to be combined with acceptable cosmetic performance [11,185].

An additional advantage of 3D printing is the possibility of structurally programmed delivery. By controlling internal architecture—such as pore size, layer thickness, and spatial distribution of components—it becomes possible to modulate diffusion pathways and release kinetics. This concept, originally developed in pharmaceutical manufacturing, enables the transition from homogeneous formulations to spatially resolved systems with locally optimised functionality.

Although still emerging, these technologies illustrate a broader movement towards digitalisation and personalisation in cosmetology. Hydrocolloid-based printable systems provide a platform that links formulation science with digital design, enabling next-generation cosmeceuticals in which both composition and geometry contribute to final product performance [184,185].

## 6. Translational Barriers, Digital Integration, and Future Directions

### 6.1. Bioelectronic Integration (Smart Patches)

Hydrocolloid-based delivery systems are increasingly converging with nanoelectronics, giving rise to flexible hydrogel patches that combine sensing, communication, and on-demand actuation while retaining the soft, hydrated, and conformable properties of the underlying matrix [201,202].

For smart patches, the hydrocolloid is only one part of a larger system. Translation also depends on sensor calibration, signal drift in biological fluids, stable adhesion during movement, power supply, data handling, actuator reliability and safe triggering of release. A responsive matrix therefore needs to be validated together with the sensing and actuation layer, not only as an isolated hydrogel.

In wound care and drug delivery, such devices can monitor local temperature, pH, glucose, uric acid, or inflammatory markers and then modulate release or trigger external intervention through a closed-loop workflow. Because hydrogel substrates are soft, hydrated, and adhesive, they provide a more compatible skin interface than rigid electronics and can be worn for prolonged periods. Future medical applications include integrated wound platforms, biohybrid interfaces, and 4D-bioprinted systems capable of real-time therapeutic adaptation [203].

Although the cosmetic use of fully closed-loop patches is less mature, the same technological logic is already relevant to personalised skincare. Printable hydrogel facial systems, hydrocolloid sheet masks, and other conformal epidermal platforms can be tailored to different skin regions and functional goals, and they therefore represent a plausible cosmetic extension of smart wearable hydrogel technology [184,185,204].

### 6.2. Regulatory Barriers and Scalability

The fundamental challenge for global implementation is standardising quality during scale-up and transfer to GMP-compatible manufacturing. Due to the natural origin of hydrocolloid materials, e.g., algae, animal chitin or plant extracts, there is substantial molecular variability between batches, depending on geographical factors, harvest conditions, extraction route and processing history [101,165,205]. Table 4 compares the key regulatory requirements for hydrogels under FDA and EMA frameworks.

This section should avoid repeating the same variability argument and instead connect it to measurable release performance. For reviewers and regulators, the practical question is whether changes in molecular weight, degree of substitution, charge density, impurity level or sterilisation history alter swelling, modulus, degradation and API release beyond the acceptable specification window.

In practice, scalability is further complicated by purification demands, sterilisation constraints, and the need to maintain reproducible rheology, swelling behaviour, and release kinetics across industrial batches, especially in multi-component or responsive systems [165].

Solutions include early regulator engagement, standardised biopolymer sourcing, impurity and molecular-weight specifications, validated sterilisation routes and process analytical technology (PAT) for real-time quality control [101,165,205]. These measures are particularly important for hydrocolloid-rich systems whose composition may vary with biological source, extraction route or post-processing history.

### 6.3. Future Directions and Data-Driven Design

AI-assisted materials design may become increasingly useful for hydrogels with programmable degradation, tunable cross-linking patterns and multi-stimuli responsiveness [143,206,207,208]. In tumour therapy, recent work on smart responsive hydrogels combined with AI-based design illustrates how data-assisted optimisation could help select trigger combinations, anticipate release behaviour and shorten formulation screening [208]. Another important direction is the development of hydrogel platforms for skin therapeutics and personalised regenerative medicine, particularly systems that combine hydrocolloid matrices with LCST- or pH-responsive behaviour to better match local physiological conditions [200,207].

AI-assisted formulation design should be presented carefully. It can help rank candidate polymers, crosslinkers, and trigger combinations, but its value is limited by sparse, non-standardised datasets, missing negative results and inconsistent reporting of swelling, modulus, loading and release conditions. Predictive models will be credible only when trained on curated experimental datasets and validated externally.

Sustainability-driven substitution of petrochemical excipients with renewable hydrocolloids is also gaining momentum in both pharmaceutical and cosmetic formulation science, although such replacement must still be balanced against processing, purification and performance requirements [97].

### 6.4. Final Summary

The most defensible future directions are not the most complex systems by default, but the systems that combine a clear material function with measurable performance: robust hydrocolloid matrices for hydration and local retention, hybrid carriers for poorly soluble or unstable actives, enzyme- or pH-responsive systems with validated trigger windows, and wearable formats in which sensing, adhesion, and release are tested as one integrated platform.

Hydrocolloid-based and hydrocolloid-dominant hybrid systems offer a useful design space because they combine hydration, bioadhesion, processability, biological familiarity and tunable network architecture. Their most realistic near-term impact is likely to be in topical and transdermal systems, wound dressings, injectable local depots, personalised hydrogel formats, and selected oral systems, where pH, mucus interaction or microbial degradation can be exploited.

The main bottlenecks are not conceptual but translational: variable biological sourcing, limited mechanical robustness, burst release, low loading of highly hydrophobic APIs, sterilisation-induced degradation, incomplete long-term stability data and uncertain classification of responsive combination products. These limitations should be addressed through standardised raw-material specifications, quantitative swelling/rheology/release benchmarks, route-specific biocompatibility testing and early regulatory alignment.

Future progress will depend on closer collaboration between polymer chemistry, formulation science, pharmacology, cosmetic science, bioelectronics, advanced manufacturing and data-assisted materials design. The most convincing next-generation systems will be those that demonstrate not only responsiveness in vitro, but also reproducible manufacturing, clear clinical or cosmetic benefit, acceptable safety margins and evidence that the hydrocolloid matrix adds functionality beyond conventional excipient behaviour.

## Figures and Tables

**Table 1:** Compact comparison of selected hydrocolloids used in delivery systems.

Hydrocolloid Class	Key Chemistry/Charge	Main Delivery Role	Main Limitation *	Ref.
Alginate	Anionic carboxylated M/G blocks	Ionotropic beads; oral protection; encapsulation	Ion exchange; burst release if loosely cross-linked	[23,24,25,26,27,65]
Pectin	Anionic galacturonic-acid backbone	pH-assisted and colon-targeted matrices	Esterification and source variability affect gelation	[60,66,67]
Chitosan	Cationic aminopolysaccharide	Mucoadhesion; wound care; absorption enhancement	pH-dependent solubility; DD/MW variability	[26,41,42,68,69,70,71,72]
Hyaluronic acid	Anionic ECM-related glycosaminoglycan	Dermal delivery; injectables; microneedles	Enzymatic degradation; cross-linker safety; cost	[47,48,73,74,75,76,77,78,79,80]
Dextran	Neutral microbial glucan	Drug conjugates; injectable dynamic networks	Often requires chemical modification for responsiveness	[32,38,81]
Carrageenan/fucoidan	Sulfated marine polysaccharides	Ion-sensitive gels; topical and local therapy platforms	Sulfation pattern and batch variability are critical	[8,28,29,30,82,83]
Gellan gum	Anionic bacterial polysaccharide	Ion-triggered in situ gels; ocular/parenteral delivery	Ion sensitivity and gel brittleness may limit use	[33,34]
Collagen/gelatin	Amphoteric protein hydrocolloids	Wound dressings; ECM-like scaffolds	Source, immunogenicity and sterilisation sensitivity	[49,51,84]
Cellulose derivatives	Neutral or anionic substituted cellulose	Sustained-release tablets; films; patches	Mainly passive; limited intrinsic responsiveness	[54,55,56,57,58]
LBG/galactomannans	Neutral plant seed polysaccharides	Rheology control; colon-oriented matrices	Microbiota-dependent degradation; weaker gel strength	[59,85]

* Note: mechanical modulus, swelling ratio, loading efficiency, release duration and degradation time are formulation-specific and should be discussed only when values are available from directly comparable studies.

**Table 2:** Compact decision matrix for stimulus-responsive hydrocolloid-based systems.

Trigger	Main Matrix Response	Specificity/Speed	Main Translational Issue	Refs.
pH	Ionisation; swelling/deswelling	Medium–high/medium	Limited selectivity in complex tissues	[20,51,90,103,107,108,110,111,112,143,144]
Enzyme	Backbone or linker cleavage; erosion	High/slow–medium	Enzyme levels vary between patients and sites	[61,113,114,115,116,117]
ROS/redox	Cleavable bonds; network loosening	Medium–high/medium	Risk of off-target oxidative/reductive activation	[91,118,119,120,121,122,123,124,145]
Thermo	Sol–gel transition; LCST-type collapse	Medium/fast	Often requires synthetic or hybrid segments	[18,124,125,126,127,128]
Magnetic/light/US	Local heating, permeability change or disruption	High spatial control/fast	Device, irradiation and nanocomponent burden	[16,17,36,129,130,131,146]
Multi-responsive	Sequential or logic-gated response	High/variable	High formulation and regulatory complexity	[16,35,64,91,132,133,134,135,136,146]

**Table 3:** Compact function-evidence matrix for cosmetic and cosmeceutical hydrocolloid platforms.

Platform	Main Cosmetic Role	Typical Actives/Formats	Evidence Limitation	Refs.
HA hydrogels/masks	Hydration; skin residence	Retinol, peptides, antioxidants; gels/masks/skin boosters	Often short-term hydration or formulation endpoints	[50,179,180]
Chitosan films/gels	Stabilisation; bioadhesion	Retinoids, polyphenols, plant extracts	pH-dependent solubility; limited clinical data	[10,181,182,183]
Alginate/carrageenan masks	Hydration; soothing; printability	Humectants, botanical extracts; printable masks	Mostly formulation and early-use studies	[11,184]
Cellulose derivatives	Rheology; prolonged residence	Vitamins, botanical extracts; gels/patches	Mainly passive matrix function	[7,8,185]
Dextran/hybrid nanogels	Nanocarrier-assisted deposition	Curcumin, gamma-oryzanol, lipophilic antioxidants	Complex formulation; limited clinical translation	[87,186,187]
Collagen/ECM-like gels	Conditioning; cosmetic-regenerative crossover	Peptides, growth-supportive actives; gels/masks	Regulatory/claim boundary; mixed evidence	[52,180]

**Table 4:** Compact FDA/EMA-oriented translational considerations for hydrocolloid-based products.

Issue	Regulatory Relevance	Practical Implication	Recommended Control	Refs.
Product classification	Device, drug, biologic or combination product depends on primary mode of action	Development pathway and evidence package may change	Clarify intended use and classification early	[101,102,165,175,176]
Source variability/CMC	Natural polymers require raw-material specification and process control	Batch changes may alter rheology, swelling and release	Define MW, DD/M/G ratio, purity and impurity limits	[101,165,205]
Sterility and microbiology	Sterilisation must match route and product risk	Sterilisation may damage network structure or API	Validate sterilisation while monitoring gel function	[101,165,205]
Biocompatibility/degradation	Cross-linkers, additives and degradation products require justification	Local tolerance and long-term safety may limit translation	Test extractables, degradation profile and local response	[95,96,101,165]
Clinical evidence and claims	Evidence must support the final therapeutic or cosmetic claim	Overstated claims increase regulatory risk	Align endpoints with intended use and product class	[100,101,164,173,175]
Scale-up and shelf life	GMP transfer requires reproducible performance over time	Drying, storage and transport may alter release or mechanics	Use PAT/rheology/swelling controls and stability testing	[101,165,205]

## Data Availability

No new data were created or analyzed in this study. Data sharing is not applicable to this article.

## Abbreviations

The following abbreviations are used in this manuscript:

3D	Three-dimensional
AHA	Alpha-hydroxy acid
API	Active pharmaceutical ingredient
CMC	Carboxymethylcellulose
CNC	Cellulose nanocrystals
ECM	Extracellular matrix
EMA	European Medicines Agency
EPS	Exopolysaccharides
EPR	Enhanced permeability and retention
FDA	Food and Drug Administration
FDM	Fused deposition modelling
FTIR	Fourier-transform infrared spectroscopy
GMP	Good Manufacturing Practice
GRAS	Generally Recognised As Safe
HA	Hyaluronic acid
HPMC	Hydroxypropyl methylcellulose
IBD	Inflammatory bowel disease
LCST	Lower critical solution temperature
LBG	Locust bean gum
MAAs	Mycosporine-like amino acids
MBBT	Methylene bis-benzotriazolyl tetramethylbutylphenol
MMPs	Matrix metalloproteinases
MRSA	Methicillin-resistant Staphylococcus aureus
MSCs	Mesenchymal stem cells
NIR	Near-infrared
NLCs	Nanostructured lipid carriers
OW	Oil-in-water
OTC	Over-the-counter
pHEMA	Poly(2-hydroxyethyl methacrylate)
PAT	Process analytical technology
PEG	Polyethylene glycol
PMS	Post-market surveillance
PNIPAM	Poly(N-isopropylacrylamide)
PLGA	Polylactic-co-glycolic acid
PVA	Polyvinyl alcohol
PVP	Polyvinylpyrrolidone
ROS	Reactive oxygen species
RGD	Arg-Gly-Asp
SEM	Scanning electron microscopy
SVX	Spider silk protein
TEWL	Transepidermal water loss
TiO_2_	Titanium dioxide
UV-Vis	Ultraviolet-visible spectroscopy
VEGF	Vascular endothelial growth factor
WO	Water-in-oil
